# Aneurism of the Arch of the Aorta, Cured by Rest, Restricted Diet and Iodide of Potassium and Ergot

**Published:** 1880-12

**Authors:** R. Stansbury Sutton

**Affiliations:** Fellow of the American Gynæcological Society, etc., etc.


ANEURISM OF THE ARCH OF THE AORTA, CURED BY REST, RESTRICTED DIET, AND IODIDE OF POTASSIUM AND ERGOT. By R. STANSBURY SUTTON, A.M., M.D., Fellow of the American Gynæcological Society, etc., etc.

Miss Mary Louise B., resident in the northern part of this county, aged twenty-one, is of delicate parentage, and possesses a highly nervous temperament. In the autumn of 1873 she was working in the garden, assisting in taking up the winter vegetables. After prolonged exertion in a stooping posture, she suddenly resumed an erect attitude. Instantly she was seized with violent pain in the region of the heart. This pain, in her own language, caused her to "shriek." Faintness immediately followed and she dropped from her feet. The pain gradually subsided. For six months from this date she had repeated attacks of pain and frequently fainted. On one occasion she fell from a porch and narrowly escaped death. During this time she had no medical care.

In the spring of 1873 her disease was diagnosticated as disease of the heart, and her case was looked upon as hopeless.

On the 4th of July, 1874, about nine months after the seizure in the garden, she was brought to my office. An examination revealed an aneurism of *the descending portion of the arch of the aorta.* Her face was of a purplish hue, and all the veins above the clavicle were turgescent. The aneurism was pressing hard against the second and third ribs just to the left of the sternum. Its thrill was distinct, and its impulse was readily discernible from that of the heart. My diagnosis was confirmed by Dr. Daniel Leasure, now of St. Paul, Minnesota. The prognosis was emphatically unfavorable, and Dr. Leasure expressed the opinion that the patient would scarcely survive the summer.

For a year, to the autumn of 1875, she remained in the city under my care. During this time she was secluded from all excitement, and took three times daily from 10–20 drops of fluid extract of ergot and 5 grains of potassium iodide. The ribs were now bulged up above the plane of the chest wall; the thrill was very loud, and the impulse visible in the second intercostal space. It seemed to me that the end was very near. Fortunately I decided upon another effort. She was sent to her home in the country and put to bed at absolute rest for five and a half out of every six hours—the half hour being allowed for quiet walking about her room. Her nourishment was restricted to ten ounces of material, fluid and solid together, for each twenty-four hours. The ergot and potassium iodide were continued. This regime was strictly adhered to for thirteen months.

At the end of this time I visited her at her own home. I found her in bed and reduced to almost a skeleton. An examination revealed the fact that the aneurism was cured, and the action of the heart weak and irregular and giving a distinct anæmic bruit. I put her upon drachm doses of bitter wine of iron, increased her nourishment, and gave permission for her to be up and down at will. This was in the autumn of 1876. Her cure appears to be permanent.

At a meeting of the Allegheny County Medical Society, held at Pittsburgh, June 15, 1880, this case was reported and the patient exhibited. A committee consisting of Drs. Thomas J. Gallaher, Andrew Fleming, and S. M. Stevenson was appointed to examine the patient. The committee reported that the aneurism was cured, and that the only evidence of disease at this time was a slight endocardial murmur.—*Am. Jour. of the Med. Sci.*, October, 1880.

---

THE course pursued by the Guy's Hospital nurse, in dressing, upon her own responsibility, the scalp wound of a patient who subsequently died from fracture of the skull, which she had not recognized, appears not to be, as might be supposed from the post mortem evidence at the coroner's inquest (which revealed serious injury to the back part of the head, immediately beneath the dressed wound), an isolated example of forgetfulness of duty or omission of instructions. We have before us details of the case of a patient who applied lately at another metropolitan hospital, with a scalp wound on the vertex. The wound was "clean cut," and did not extend deep enough to expose the bone; it was about an inch long; the surrounding hair had been clipped off, apparently preparatory to the application of a surgical dressing. The statement of the man, who was sober, was to the effect that he had gone to Guy's and asked to see the house surgeon or dresser, but that a nurse had informed him that he could not see that official, and immediately she began to cut the hair from around the wound. Upon this the man left and came to St. Thomas's, where he was attended to. This incident suffices to show the distressing scandal exposed at this inquest is a logical sequence of the system by which the nursing at Guy's has been made to supercede and override the responsibility of the medical staff, instead of being the docile and skilled instrument. The nurse is one of the Leicester nurses introduced by the matron as being trained in the system which she carried out at the hospital of that town prior to her appointment at Guy's Hospital. Nothing could more clearly justify the demand of the staff that the system be abolished root and branch.—*Brit. Med. Jour.*, Oct. 30, 1880.

---

GLASS HOUSES AND STONES.—The author of the Guide to European Universities, Dr. Hardwicke, of Sheffield, has been induced, by the success of his pamphlet, to reproduce the information therein conveyed in book form, together with the results of his inquiries into other systems of education, and other countries than Europe. Here the reader will find information respecting the educational bodies, examinations, and medical laws of every civilized state, and he will also come to the humiliating confession that though there exist a good many time-honored institutions in the United States, and an anxiety to put matters on a scientific footing in others, yet farther South the condition of medicine is as bad as can possibly be imagined. Bankrupt bakers, grocers, and others who have failed to make a living, afterwards start as 'doctor,' and soon get into *full practice*, by parading their names before the public, through the medium of the daily papers and street placards, with "Dr." and "M. D." attached to them, without a word being said by the authorities. All this is very humiliating, but we could show our author identical counterparts in London, Birmingham, Glasgow, and other large cities, without a word being said by the authorities. But they, not Dr. Hardwicke, are responsible for this, and we cordially thank him for his decidedly useful addition to our knowledge of medical education in other countries.—*Medical Press and Circular.*

